# Mitigating Text Dominance in Medical Vision–Language Learning for Long-Tailed Multi-Label Chest X-Ray Classification

**DOI:** 10.3390/diagnostics16213551

**Published:** 2026-10-28

**Authors:** Aqeeb Hussain, Sabeen Masood, Samera Batool, Ali Hassan, Shuja Ansari, Sanaa Ghouzali, Wadood Abdul

**Affiliations:** 1 Department of Software Engineering, Capital University of Science and Technology, Islamabad 44000, Pakistan; 2 Department of Computer Science, COMSATS University, Islamabad 44000, Pakistan; 3 Department of Computer and Software Engineering, College of Electrical and Mechanical Engineering, National University of Sciences and Technology (NUST), Islamabad 44800, Pakistan; 4 James Watt School of Engnireeing, University of Glasgow, Glasgow G12 8QQ, UK; 5 Department of Electrical and Communication Engineering, College of Engineering, UAE University, Al Ain P.O. Box 15551, United Arab Emirates; 6 LyRICA Laboratory, Meridian Team, School of Information Sciences, Rabat 10100, Morocco; 7 Department of Computer Engineering, College of Computer and Information Sciences, King Saud University, P.O. Box 51178, Riyadh 11543, Saudi Arabia

**Keywords:** chest X-ray, medical vision–language learning, multimodal learning, modality imbalance, text dominance, modality dropout, multi-label classification

## Abstract

**Background/Objectives**: Medical vision–language learning integrates radiographic images and textual information for chest X-ray (CXR) classification. However, in long-tailed multi-label CXR classification, modality imbalance can cause models to rely disproportionately on radiology reports while underutilizing visual information. This study investigates text dominance and proposes a modality-balanced framework to strengthen visual representation while preserving multimodal performance. **Methods**: Experiments used MIMIC-CXR with patient-level training, validation, and test splits of 32,942, 4024, and 3955 paired samples, respectively. Swin-Tiny and PubMedBERT served as visual and textual encoders. The proposed framework combines adaptive gated fusion, Asymmetric Loss (ASL), and training-time modality dropout. It was compared with Binary Cross-Entropy (BCE) concatenation, ASL concatenation, gated fusion, and cross-attention fusion. **Results**: The BCE baseline achieved a Macro AUROC of 0.9957 under Image + Text evaluation and 0.5804 under Image-Only evaluation, while Text-Only evaluation yielded 0.9955, indicating substantial modality disparity. Other alternatives achieved Image-Only Macro AUROC values of 0.5610–0.5720. The proposed framework improved Image-Only Macro AUROC to 0.7697 and Macro PR-AUC from 0.1814 to 0.3428 while maintaining an Image + Text Macro AUROC of 0.9958. The modality-dominance gap decreased from 0.4151 to 0.2260, a 45.6% reduction in performance disparity. Grad-CAM and Integrated Gradients provided insights into visual evidence and textual attribution, revealing reliance on explicit disease-related terminology in reports. **Conclusions**: These findings suggest that combining adaptive gated fusion, ASL, and modality dropout can improve Image-Only performance while preserving multimodal discrimination in long-tailed multi-label CXR classification.

## 1. Introduction

Chest radiography is among the most widely used imaging modalities for the detection, diagnosis, and monitoring of cardiopulmonary abnormalities. Its accessibility, relatively low acquisition cost, and routine integration into clinical workflows have made automated chest X-ray (CXR) interpretation an important area of medical artificial intelligence. Large-scale datasets such as MIMIC-CXR have accelerated this research by providing chest radiographs paired with free-text radiology reports [1]. Advances in deep learning have further expanded CXR analysis from convolutional architectures to transformer-based models such as the Swin Transformer, which captures hierarchical visual representations through shifted-window attention [2]. Despite these advances, automated CXR interpretation remains challenging because multiple thoracic findings may coexist, abnormalities may exhibit subtle or overlapping visual characteristics, and disease prevalence is highly imbalanced. Radiology reports provide another rich source of information because they describe anatomical structures, observed abnormalities, diagnostic impressions, and uncertainty associated with radiographic interpretation. The availability of paired image–report datasets has therefore encouraged medical vision–language learning, in which visual and textual representations are jointly modeled.

Domain-specific language models such as PubMedBERT provide contextual representations adapted to biomedical terminology [3]. Medical vision–language approaches have demonstrated the value of paired radiographs and reports through contrastive image–text learning, global–local alignment, and biomedical semantic modeling [4,5,6]. More recent methods have extended this direction through cross-supervised representation learning, medical knowledge-enhanced image–text learning, and multimodal disease prediction [7,8,9,10]. However, the availability of multiple informative modalities does not guarantee that a multimodal model utilizes them equally or effectively. During joint optimization, a modality providing an easier or more direct predictive pathway may dominate learning and reduce optimization pressure on the weaker pathway. Balanced multimodal learning research has demonstrated that unequal optimization between modality branches can prevent weaker modalities from being learned effectively [11]. Subsequent studies have investigated this problem through diagnostic and re-learning strategies, dynamic fusion, and gradient-based balancing mechanisms [12,13,14]. Recent investigations have further identified language or text dominance as an important form of asymmetric modality dependence in multimodal systems [15,16,17].

This problem is particularly relevant to paired CXR–report classification. Radiology reports are generated to describe findings observed in the corresponding radiographs and may therefore contain explicit or highly predictive descriptions of the same thoracic abnormalities used as classification targets. Consequently, a multimodal classifier can achieve excellent Image + Text performance primarily through its textual pathway while retaining substantially weaker visual representations. Related concerns regarding modality dominance have also been reported in radiology vision–language systems [18]. Therefore, near-saturated multimodal performance should not automatically be interpreted as evidence that both visual and textual information have been learned effectively. Missing-modality and modality-robust learning provide a related perspective. Modality dropout and incomplete-modality training strategies encourage multimodal models to preserve useful representations when one information source becomes unavailable [19,20,21,22,23]. Earlier medical imaging studies similarly demonstrated the value of learning under absent or randomly suppressed modalities [24,25]. Although such approaches are generally motivated by robustness to missing inputs, modality removal can additionally serve as a diagnostic mechanism for examining learned dependence. Evaluating the same trained model under Image + Text, Image-Only, and Text-Only conditions can reveal whether complete-input performance genuinely depends on both modalities or can be reproduced predominantly through one pathway. A second and distinct challenge arises from the long-tailed distribution of thoracic findings. Multi-label CXR datasets contain substantial differences in disease prevalence, while negative observations considerably outnumber positive examples for several findings. Conventional Binary Cross-Entropy (BCE) can therefore be influenced disproportionately by frequent labels and easily classified negative samples. Asymmetric Loss (ASL) addresses this problem through different focusing behavior for positive and negative examples [26]. Recent CXR studies have continued to investigate asymmetric optimization, prototype-based learning, and other strategies for long-tailed multi-label classification [27,28,29]. Multimodal CXR learning can consequently involve two related but distinct optimization problems: label imbalance, arising across thoracic findings, and modality imbalance, arising from unequal predictive strength between radiographic and textual inputs. This study investigates these challenges using a medical vision–language framework based on Swin-Tiny and PubMedBERT. Importantly, rather than assuming that imbalance-aware loss or more sophisticated fusion inherently produces balanced modality utilization, a controlled sequence of multimodal configurations is examined. These include concatenation with BCE, concatenation with ASL, adaptive gated fusion with ASL, cross-attention fusion with ASL, and the proposed modality-balanced configuration combining adaptive gated fusion, ASL, and training-time modality dropout. Adaptive gating enables learned weighting of projected visual and textual representations [30], whereas modality dropout explicitly exposes the model to reduced-modality conditions during optimization. This experimental design examines whether changes in the loss function or fusion architecture are sufficient to reduce text dominance and whether explicit modality-balanced training offers further benefits. The evaluation distinguishes between predictive performance and the extent to which the model utilizes each modality. Each multimodal configuration is evaluated under Image + Text, Image-Only, and Text-Only conditions, alongside an independently trained Image-Only model as a visual control. This comparison helps determine whether poor Image-Only performance after multimodal training results from limited discriminative information in the chest X-ray (CXR) or from insufficient use of visual information when the model is jointly trained with a stronger textual pathway. The independently trained Image-Only model provides a reference for examining these possibilities.

The experimental results reveal pronounced text dominance across conventional and alternative multimodal configurations. Concatenation with BCE achieved a test Macro AUROC of 0.9957 with Image + Text input and 0.9955 with Text-Only input, but performance decreased to 0.5804 when text was removed. Importantly, replacing BCE with ASL or employing adaptive gated and cross-attention fusion did not independently resolve this behavior. These configurations achieved Macro AUROC values of 0.9961–0.9978 when both image and text were available, whereas their Image-Only Macro AUROC values remained between 0.5610 and 0.5720. Thus, improved complete-input discrimination or more expressive fusion did not necessarily translate into stronger preservation of visual information. Meanwhile, the proposed modality-balanced framework improved Image-Only Macro AUROC to 0.7697 while achieving an Image + Text Macro AUROC of 0.9958. The Macro AUROC modality-dominance gap decreased from 0.4151 for conventional concatenation to 0.2260, corresponding to an approximately 45.6% reduction in measured asymmetric dependence. Furthermore, the independently trained visual model achieved a test Macro AUROC of 0.7896, demonstrating that substantial discriminative information was available from the CXR modality itself. The proposed multimodal visual pathway therefore approached the independently optimized visual performance, although Text-Only performance remained close to complete-input performance. The results consequently support mitigation rather than elimination of text dominance.

The novelty of this study lies in its problem-specific investigation and mitigation of visual-modality underutilization in multimodal chest X-ray (CXR)–report classification. Rather than introducing a new standalone fusion operator, loss function, or modality-dropout mechanism, this work integrates adaptive gated fusion, training-time modality dropout, and Asymmetric Loss (ASL) within a unified framework to investigate whether the visual pathway can retain greater discriminative capability without substantially sacrificing complete-input performance. A distinguishing aspect of this study is its evaluation strategy, which examines complete-input performance alongside Image-Only and Text-Only inference and quantifies modality dependence through a modality-dominance gap. This evaluation makes it possible to identify weaknesses that may remain concealed by near-saturated multimodal classification metrics. Furthermore, controlled comparisons of loss and fusion configurations investigate whether ASL or alternative fusion mechanisms alone are sufficient to preserve visual discriminative capability. The study therefore contributes a problem-specific framework and empirical analysis for evaluating and mitigating modality dominance in CXR–report classification, rather than claiming novelty for its individual established components.

The main contributions of this study are as follows:

Systematic evaluation of modality dependence: Complete-input, Image-Only, and Text-Only evaluations are used to reveal asymmetric image–text dependence that may remain concealed by high multimodal classification performance.

Comparative evaluation of loss and fusion configurations: Concatenation with Binary Cross-Entropy (BCE), concatenation with ASL, adaptive gated fusion with ASL, and cross-attention fusion with ASL are evaluated to investigate whether imbalance-aware loss or alternative fusion mechanisms alone are sufficient to preserve visual discriminative capability.

Assessment of visual-pathway underutilization: An independently optimized Image-Only model provides a visual reference for interpreting the discriminative capability available from CXR images and assessing how much visual performance is retained by the multimodal model following text removal.

A modality-balanced training framework: The proposed framework combines training-time modality dropout, adaptive gated fusion, and ASL to mitigate text dominance while addressing the long-tailed 14-label classification task. The contribution lies in the integration and evaluation of these mechanisms for the stated problem, rather than in presenting them as individually new techniques.

Quantification of visual-pathway recovery: The proposed configuration increases Image-Only Macro AUROC from 0.5804 to 0.7697 while maintaining Image + Text Macro AUROC at 0.9958. The modality-dominance gap decreases from 0.4151 to 0.2260, representing an approximately 45.6% reduction. These results quantify the improvement in visual-pathway performance under modality removal in the evaluated setting.

The paper is divided into six parts. Section 2 reviews works related to Chest X-ray classification, medical vision–language models, text dominance, modality imbalance, and robustness to missing modalities to motivate this work. In Section 3, explanations of the dataset, model components, fusion methods, proposed training framework, and experimental setup are provided. Section 4 presents the results of the experiments, including results for the Image-Only and multimodal performance, fusion configuration comparison, modality ablations, and individual disease label results. These results are discussed and interpreted in Section 5, which also explores the limitations of the proposed approach. Section 6 concludes the study and offers some ideas for additional research.

## 2. Related Work

There have been several advancements in the field of CXR classification, moving beyond traditional CNN models to transformer networks and multimodal approaches. The Swin Transformer is a self-attention-based model that learns hierarchical visual representations by using shifted-window self-attention [2]. For CXR classification, transformer-based and hybrid models have been employed recently, such as GViT using Swin Transformer V2 [31] and multi-view attention and ensemble learning [32]. But the classification of CXR is still difficult since the labels of diseases are long-tailed and some of the abnormalities are rarer than others. Many of the negative labels also pose problems with training and optimizing the model. Positive-negative imbalance has been tackled via asymmetric focusing [26], and later studies explored long-tailed multi-label learning with LM-CLIP [27], prior-aware prototype-based contrastive learning [28], alternative loss and architecture designs [29], and multi-center long-tailed evaluation with CXR-LT [33]. Currently, paired images and reports have led to medical vision–language learning. Later, ConVIRT [4] and GLoRIA [5] introduced representation learning of image–text from X-rays and their associated reports, and the following works applied cross-supervision [7] or grounded knowledge-enhanced pre-training [8] or unpaired image–text learning with MedCLIP [34] or knowledge-enhanced X-ray representation learning with MedKLIP [35]. Multimodal methods have then been used for the classification of lung diseases [9], retrieval and disease prediction of CXR [36], and for robustness and generalization studies [10], while recent reviews highlight the broader development and remaining challenges of medical multimodal models [37]. Despite these advances, most studies emphasize representation quality, image–text alignment, or complete-input predictive performance rather than determining whether both modalities remain independently discriminative after joint optimization. This distinction is important because multimodal learning does not inherently guarantee balanced utilization of its constituent modalities [38]. Stronger modalities can dominate joint optimization [11], resulting in under-optimized weaker pathways [12], and several approaches have consequently explored dynamic fusion [13], gradient reconciliation [14], and gradient-guided distillation [39].

Language dominance and competition between modalities have been reported in broader multimodal models [15,16,17], with related concerns also emerging in medical applications, including radiology-focused modality dominance [18] and multimodal clinical prediction [40]. Research on missing modalities addresses a related challenge: maintaining predictive performance when one or more information sources are unavailable. Existing approaches include modality dropout [19], learning with severely missing modalities [20], unbiased missing-modality learning [21], MissModal [22], and broader frameworks for missing-modality scenarios [23]. Related strategies have also been explored in medical imaging through random modality dropout [24], cross-modality representation learning [25], and methods designed to encourage networks to use information from multiple views [41]. Fusion architectures also differ in how modality information is integrated; conventional concatenation provides direct feature combination, gated multimodal units learn modality-specific information flow [30], dynamic fusion adapts integration according to modality characteristics [13], and cross-attention enables explicit cross-modal interaction. Nevertheless, greater fusion complexity does not necessarily prevent a highly predictive modality from dominating optimization, while ASL primarily addresses label imbalance rather than modality imbalance [26,27,28,29]. These findings motivate evaluating modality utilization separately from conventional complete-input multimodal performance.

Although previous studies have investigated medical image–text representation learning [4,5,6,7,8,34,35], multimodal CXR prediction [9,10,36], long-tailed classification [26,27,28,29,33], balanced multimodal optimization [11,12,13,14,39], missing-modality robustness [19,20,21,22,23,24,25], and text or modality dominance [15,16,17,18,40], limited attention has been given to systematically determining whether a high-performing paired CXR–radiology-report classifier preserves disease-discriminative capability in its visual pathway when the textual modality provides a substantially stronger predictive signal. Complete Image + Text performance alone cannot distinguish complementary multimodal utilization from predominant reliance on report information, while poor Image-Only performance after multimodal training cannot establish visual underutilization unless the discriminative capability of an independently optimized visual model is also known. Furthermore, it remains unclear whether label-imbalance optimization through ASL or increasingly expressive fusion through adaptive gating and cross-attention is sufficient to reduce asymmetric modality dependence. This study investigates text dominance through controlled comparisons of concatenation with BCE, concatenation with ASL, adaptive gated fusion with ASL, cross-attention with ASL, and a modality-balanced framework combining adaptive gated fusion, ASL, and training-time modality dropout. Each multimodal configuration is assessed under Image + Text, Image-Only, and Text-Only conditions. An independently trained Image-Only model serves as a reference for evaluating the discriminative capability of chest radiographs without competition from textual information. These comparisons allow modality dominance to be quantified and examine whether modality-balanced training improves visual discrimination while maintaining strong performance when both image and text are available. The comparison of existing studies with the proposed framework are presented in Table 1.

## 3. Methods

### 3.1. Dataset and Experimental Cohort

The experiments used the MIMIC-CXR database, which contains de-identified chest radiographs and their corresponding free-text radiology reports [1]. The associated structured labels were obtained from the MIMIC-CXR-JPG resource [42]. Only frontal chest radiographs with an associated report and valid target-label information were retained. The final experimental cohort contained 40,921 paired image–report samples. The classification task included 14 thoracic observations: Atelectasis, Cardiomegaly, Consolidation, Edema, Enlarged Cardiomediastinum, Fracture, Lung Lesion, Lung Opacity, No Finding, Pleural Effusion, Pleural Other, Pneumonia, Pneumothorax, and Support Devices. These observations follow the CheXpert-style label formulation commonly used for chest radiograph analysis [43]. The structured labels were obtained from the MIMIC-CXR-JPG resource [42]. These labels represent the 14 thoracic observations used in the classification task. The handling of uncertain observations is described below. Because the study uses both radiology reports and structured labels, the relationship between the report text and the target-label generation process is relevant to interpreting Text-Only performance.

To reduce the risk of patient-level information leakage, the dataset was partitioned using patient identifiers rather than individual image–report samples. The resulting partitions contained 32,942 training samples, 4024 validation samples, and 3955 independent-test samples. The corresponding patient counts were 12,503, 1563, and 1563, respectively. The validation set was used for model selection and class-specific threshold determination, whereas the independent test set was reserved for final evaluation. The dataset partitions and their respective roles in model development and evaluation are summarized in Table 2.

Uncertain observations were handled using the U-Ignore strategy. Labels marked as uncertain were excluded from the corresponding loss and metric calculations rather than being converted automatically to either positive or negative targets. This treatment prevents ambiguous observations from introducing potentially incorrect supervision.

Relationship Between Report Text and Reference Labels. The classification targets were obtained from the structured labels provided by MIMIC-CXR-JPG. Because the proposed framework uses radiology report text as an input modality, the report may contain explicit or semantically equivalent descriptions of the thoracic findings represented by the target labels. Consequently, the high Text-Only Macro AUROC should be interpreted with caution, as it may partly reflect the availability of label-related information in the report text rather than independent visual–textual reasoning. The extent of this overlap depends on the report content and the procedure used to generate the reference labels.

### 3.2. Image Preprocessing and Visual Encoder

**Figure 1:**
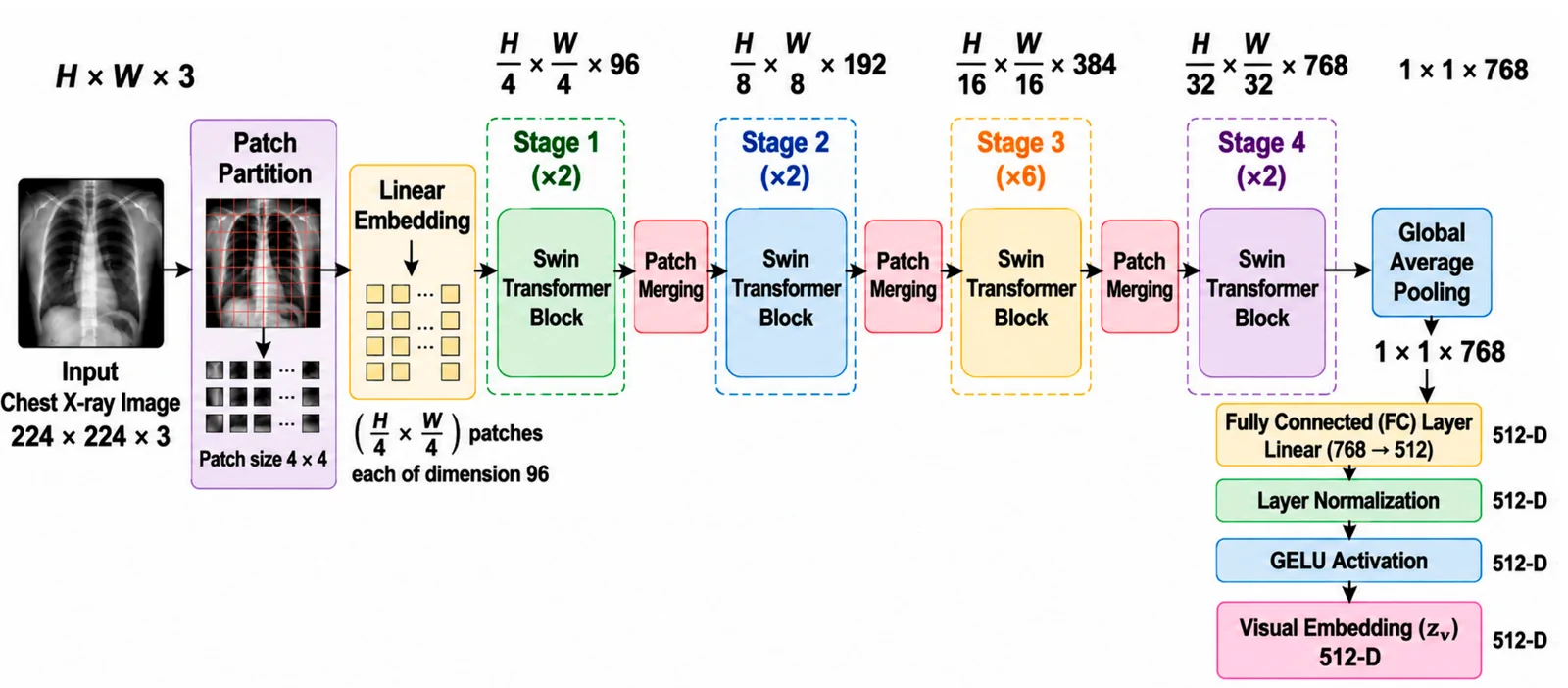
Architecture of the Swin-Tiny visual encoder and visual projection pathway.

All chest radiographs were resized to 224 × 224 pixels and transformed according to the preprocessing requirements of the visual backbone. Training-time augmentation was applied consistently across experiments and restricted to transformations that preserved clinically meaningful radiographic structure. The visual branch used Swin-Tiny, based on the shifted-window hierarchical Transformer architecture [2]. Swin Transformer progressively constructs multi-scale image representations while limiting self-attention computation to local windows and exchanging information through shifted-window operations. For the ith input radiograph I_i_, visual feature extraction was defined in Equation (1).

(1) $$h_{i}^{v}=E^{v}\left(I_{i}\right)$$

where i denotes the sample index, E^v^ is the Swin-Tiny visual encoder, I_i_ is the radiograph of sample i, and $h_{i}^{v}$ is the extracted visual feature representation. The encoder output was subsequently projected into a common latent representation as shown in Equation (2).

(2) $$z_{i}^{v}=\phi^{v}\left(W^{v}h_{i}^{v}+b^{v}\right)$$

where W^v^ and b^v^ are learnable visual projection parameters, ϕ^v^ denotes the visual projection transformation, and $z_{i}^{v}$ is the resulting projected visual representation. The detailed architecture of the visual encoding and projection pathway is illustrated in Figure 1. Swin-Tiny was selected as the visual encoder because its hierarchical architecture progressively extracts visual representations at multiple spatial scales, while shifted-window self-attention limits computational complexity compared with global self-attention. These properties make it a practical choice for extracting radiographic features under the computational constraints of this study. Its visual representations provide the image-based input to the proposed adaptive gated fusion module, allowing the contribution of radiographic information to be evaluated under multimodal and Image-Only conditions.

### 3.3. Text Preprocessing and Textual Encoder

**Figure 2:**
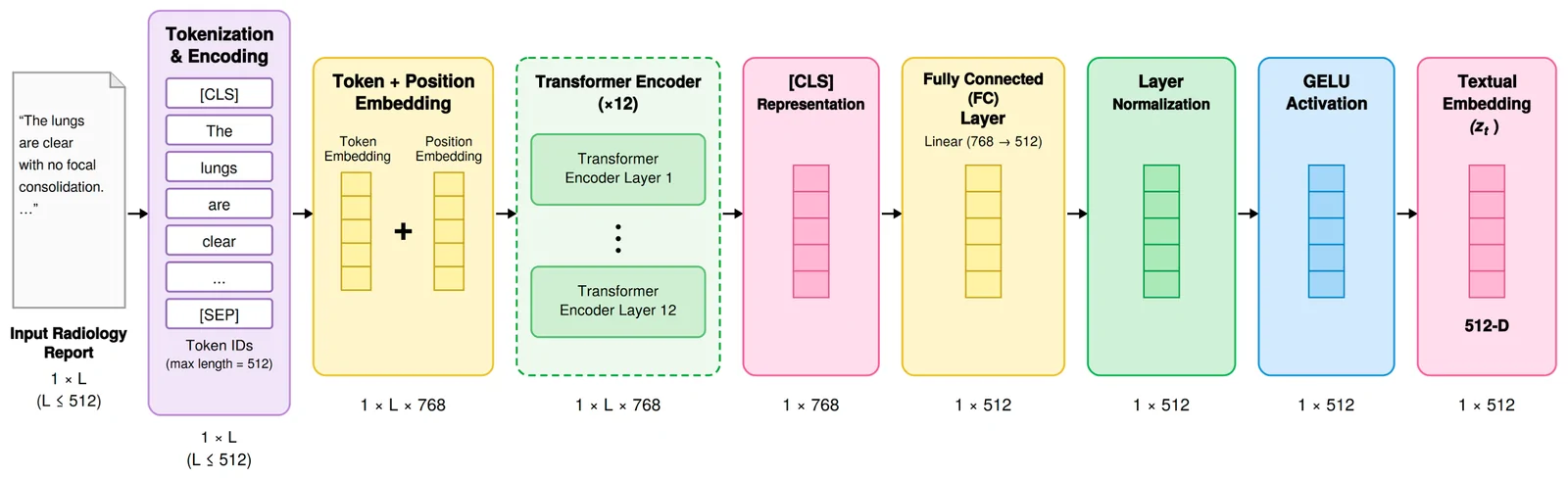
Architecture of the PubMedBERT textual encoder and textual projection pathway.

Free-text radiology reports associated with the selected examinations were processed using the tokenizer corresponding to PubMedBERT, a domain-specific biomedical language model [3]. Reports were tokenized to a maximum sequence length of 512 tokens, with padding or truncation applied where necessary. Token identifiers and attention masks were provided to the textual encoder. For the radiology report *T_i_* associated with sample *i*, contextualized textual encoding is defined in Equation (2).

(3) $$H_{i}^{t}=E^{t}\left(T_{i}\right)$$

where *E^t^* denotes the PubMedBERT textual encoder, and $H_{i}^{t}$ represents the sequence of contextualized token embeddings obtained from report *T_i_*. A global report representation was obtained from the first contextualized token representation as shown in Equation (4).

(4) $$h_{i}^{t}=H_{i,0}^{t}$$

where $H_{i,0}^{t}$ denotes the contextualized representation at the first token position and hit is the resulting global textual representation for sample *i*. The global report representation was then projected into the same latent feature space as the visual representation, as shown in Equation (5).

(5) $$z_{i}^{t}=\phi^{t}\left(W^{t}h_{i}^{t}+b^{t}\right)$$

where *W^t^* and *b^t^* are learnable textual projection parameters, *ϕ^t^* denotes the textual projection transformation, and $z_{i}^{t}$ is the projected textual representation. The detailed architecture of the textual encoding and projection pathway is illustrated in Figure 2.

PubMedBERT was selected as the textual encoder because it was pretrained on biomedical literature and can represent domain-specific biomedical terminology relevant to medical reports. Its contextualized representations provide the textual input to the proposed adaptive gated fusion module. Although this domain-specific pretraining is beneficial for processing biomedical language, radiology-report terminology may also overlap with the target labels. Therefore, the resulting textual representations must be evaluated alongside Image-Only and complete-input performance to investigate text dominance and visual-pathway retention. Both modalities were mapped to a common 512-dimensional latent space as shown in Equation (6).

(6) $$z_{i}^{v},z_{i}^{t}\in R^{512}$$

where $R^{512}$ denotes the shared 512-dimensional real-valued feature space used for multimodal fusion. Domain-specific biomedical language models provide representations well suited to biomedical terminology [3], while previous medical vision–language studies have demonstrated the effectiveness of learning from paired radiographs and reports [4,5,6,7,8,34,35].

### 3.4. Standalone Unimodal Controls

Independent Image-Only and Text-Only models were used to characterize the predictive capability available from each modality when optimized separately. The standalone Image-Only model used Swin-Tiny without access to report information. This experiment was particularly important for determining whether weak Image-Only performance after multimodal training reflected limited visual information or underutilization of the visual pathway during joint optimization. The standalone Text-Only model used PubMedBERT without radiographic input and provided a corresponding estimate of the predictive information available from the radiology reports. To examine whether the strong predictive capability of the textual pathway was specific to the selected language encoder, an additional Text-Only encoder ablation was performed using BioClinicalBERT under the same patient-wise data partitioning and optimization protocol. This comparison was used as a control to determine whether the observed text-dominant behavior could be attributed specifically to PubMedBERT or was consistently present across alternative biomedical language encoders.

### 3.5. Controlled Multimodal Experimental Design

To separate the effects of fusion architecture, imbalance-aware optimization, and modality-balanced training, five multimodal configurations were considered while keeping the principal experimental protocol fixed. The five controlled multimodal configurations used to isolate the effects of fusion strategy, imbalance-aware optimization, and modality-balanced training are summarized in Table 3.

The same Swin-Tiny visual encoder, PubMedBERT textual encoder, 512-dimensional projection space, patient-wise split, image resolution, maximum text length, batch size, uncertainty handling, and evaluation protocol were retained across the controlled multimodal experiments. This design permits comparison between a conventional multimodal classifier and progressively modified configurations without changing the underlying data or encoder families.

### 3.6. Conventional Concatenation Fusion

The conventional baseline combined the projected image and text representations by feature concatenation as shown in Equation (7).

(7) $$z_{i}^{\mathrm{cat}}=\left[z_{i}^{v};z_{i}^{t}\right]$$

where [;] denotes vector concatenation and zicat represents the joint multimodal feature vector for sample i. Since each modality representation contains 512 features, the concatenated representation contains 1024 features. Multi-label predictions were then generated from the concatenated representation as shown in Equation (8).

(8) $${\hat{p}}_{i}=\sigma \left(f_{c}\left(z_{i}^{\mathrm{cat}}\right)\right)$$

where f_c_ denotes the learnable multi-label classification head, σ is the element-wise sigmoid function, and p_i_ contains the predicted probabilities for the 14 thoracic observations.

Experiment A used masked Binary Cross-Entropy, whereas Experiment B retained exactly the same concatenation architecture but replaced BCE with ASL. This control enables the effect of the imbalance-aware objective to be separated from changes in the fusion architecture.

### 3.7. Cross-Attention Fusion

Cross-attention was evaluated as an interaction-based alternative to direct concatenation. Unlike concatenation, which combines already summarized representations, cross-attention permits features originating from one modality to condition the representation learned from the other modality. Visual information was updated using the textual representation as shown in Equation (9).

(9) $${\tilde{z}}_{i}^{v}=\mathrm{MHA}\left(Q=z_{i}^{v},K=z_{i}^{t},V=z_{i}^{t}\right)$$

where MHA denotes multi-head attention, Q, K, and V denote the query, key, and value inputs, respectively, and $z_{i}^{v}$ is the text-conditioned visual representation. Conversely, textual information was updated using visual information as shown in Equation (10).

(10) z~i t= MHAQ = zit,K =ziv,V =ziv

where $z_{i}^{t}$ denotes the visual-conditioned textual representation for sample i. The resulting cross-modal features were combined and passed to the classifier. Cross-attention therefore served as a strong fusion control for determining whether more explicit cross-modal interaction alone could reduce the observed dependence on text.

### 3.8. Adaptive Gated Fusion

Adaptive gated fusion was used to learn the relative contributions of visual and textual representations instead of treating them identically. Gated multimodal fusion has previously been introduced as a mechanism for controlling information flow from heterogeneous modalities [30]. The gating vector was generated from the concatenated projected representations as shown in Equation (11).

(11) $$g_{i}=\sigma \left(f_{g}\left(\left[z_{i}^{v};z_{i}^{t}\right]\right)\right)$$

where f_g_ denotes the learnable gating network, σ is the sigmoid function, and g_i_ determines the relative contribution assigned to the two modalities for sample i. The gated multimodal representation was then computed as shown in Equation (12).

(12) $$z_{i}^{g}=g_{i}⊙z_{i}^{v}+\left(1-g_{i}\right)⊙z_{i}^{t}$$

where ⊙ denotes element-wise multiplication and $z_{i}^{g}$ is the adaptively fused representation.

Experiment D used this adaptive gate with ASL but without modality dropout. This configuration served as an important component control because it tests whether adaptive gating alone is sufficient to address asymmetric modality dependence.

### 3.9. Proposed Modality-Balanced Framework

The proposed model retained the adaptive gated fusion architecture while incorporating training-time modality dropout to mitigate excessive reliance on the highly predictive textual pathway while preserving multimodal discriminative performance. The complete framework comprised a Swin-Tiny visual encoder for extracting radiographic representations and a PubMedBERT text encoder for encoding the associated radiology reports. The resulting visual and textual representations were transformed into a common 512-dimensional embedding space through modality-specific projection layers. During training, modality dropout was applied before fusion to encourage the model to learn predictive information from both modalities rather than relying predominantly on the textual representation. The resulting modality representations were subsequently integrated using adaptive gated fusion, and the final multi-label predictions were optimized using Asymmetric Loss (ASL).

**Figure 3:**
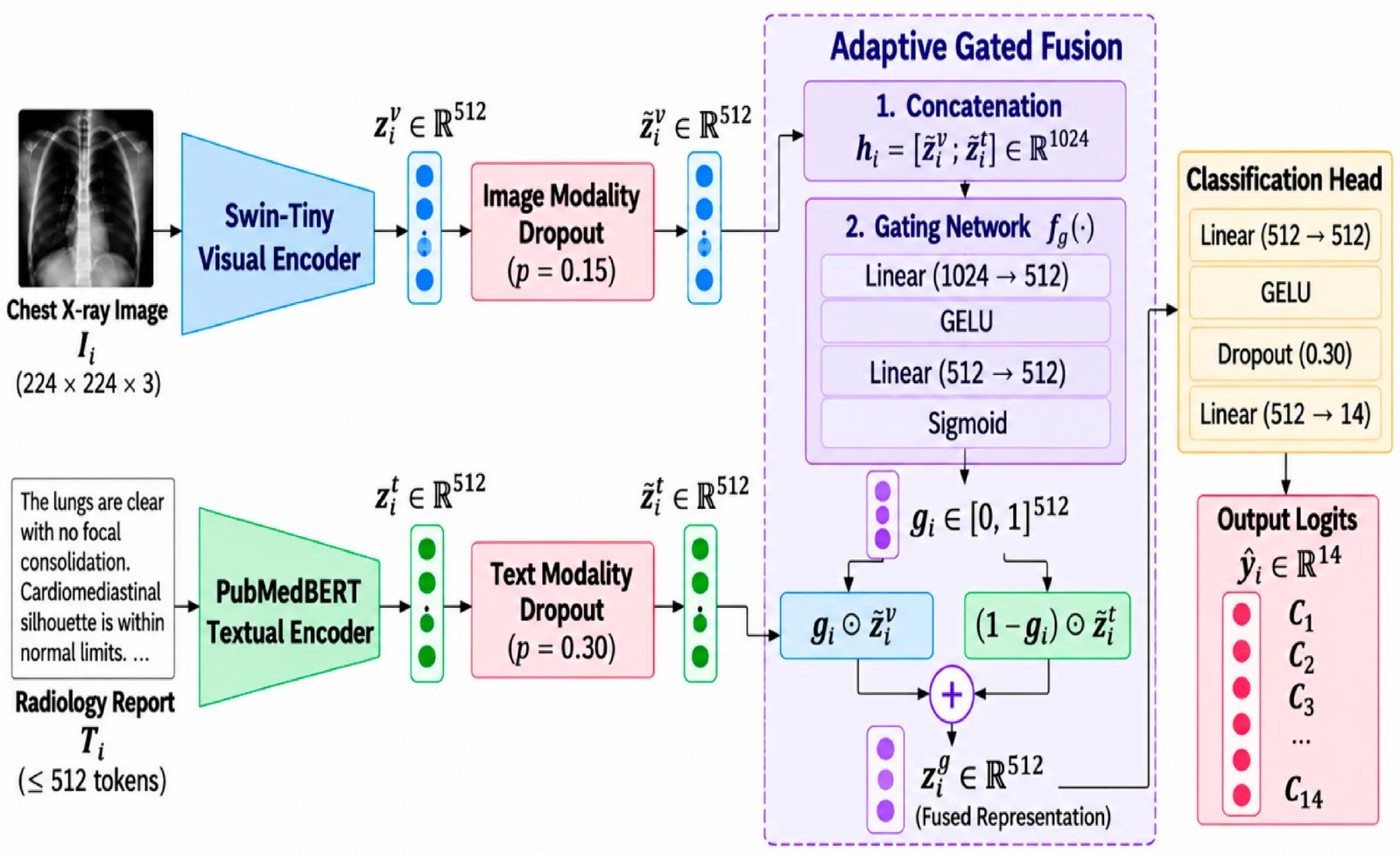
Architecture of the proposed modality-balanced vision–language framework.

The overall architecture of the proposed modality-balanced framework is illustrated in Figure 3.

During ordinary multimodal training, both modalities are continuously available. If textual information provides a much easier path to the target, optimization can achieve low loss without developing equally discriminative visual representations. Modality-dropout approaches have been investigated as a means of improving robustness and preventing excessive co-adaptation between information sources [19,24]. Training-time modality dropout was applied as shown in Equation (13).

(13) $${\tilde{z}}_{i}^{v}=m_{i}^{v}z_{i}^{v},\;\;{\tilde{z}}_{i}^{t}=m_{i}^{t}z_{i}^{t}$$

where $m_{i}^{v}$ and $m_{i}^{t}$ are modality-availability variables controlling the visual and textual representations, respectively, and the tildes denote the representations after modality dropout. For the primary proposed configuration, the image-modality dropout probability was set to 0.15, whereas the text-modality dropout probability was set to 0.30. The larger dropout probability assigned to the textual pathway was intended to impose stronger regularization on the dominant modality and encourage greater utilization of visual information. To assess whether the observed modality-balancing behavior was specific to these probabilities, a post hoc sensitivity analysis was additionally conducted using alternative asymmetric modality-dropout configurations, including a reversed asymmetric setting and a nearby probability setting. These additional configurations were used to examine the robustness of the observed modality-dependence pattern and were not used to retrospectively select the primary 0.15/0.30 configuration. The corresponding results are presented in Section 4. At inference, modality dropout was disabled; therefore, complete Image + Text evaluation supplied both modality representations without stochastic removal. Training-time modality dropout should be distinguished from evaluation-time modality ablation. The former modifies the training conditions to encourage more robust utilization of the available modalities, whereas the latter deliberately removes one modality after training to quantify the model’s dependence on visual or textual information. Inference Protocol under Modality Removal. The Image + Text, Image-Only, and Text-Only conditions were evaluated using the same trained multimodal checkpoint, without retraining or fine-tuning between conditions. For Image + Text inference, both the chest X-ray image and its corresponding radiology report were provided. For Image-Only inference, the textual modality was removed, whereas for Text-Only inference, the image modality was removed. This evaluation protocol measures the predictive capability retained by the trained multimodal model when one modality is unavailable. Accordingly, the Image-Only and Text-Only results should not be interpreted as the performance of independently optimized unimodal models. Removing a modality at inference changes the information available to the trained model without adapting its parameters to the altered input condition. Therefore, differences in performance across the three conditions provide evidence of modality dependence and robustness to missing inputs, rather than a direct comparison of the maximum achievable performance of separate unimodal systems. To quantify the disparity between the predictive capabilities of the textual and visual modalities, we define the modality-dominance gap as the difference between their unimodal Macro AUROC scores:

Δ*_MD_* = AUROC_Text_ − AUROC_Image_

where AUROC_Text_ and AUROC_Image_ denote the Macro AUROC scores obtained under Text-Only and Image-Only inference, respectively, using the same trained multimodal checkpoint. A larger positive gap indicates a greater disparity in favor of the textual modality, whereas a gap closer to zero indicates more comparable unimodal ranking performance. Macro AUROC is used because it assesses discrimination across classification thresholds and is less directly dependent on a fixed decision threshold than F1. However, the modality-dominance gap measures differences in unimodal predictive performance rather than complementary information use directly. A smaller gap alone does not establish that the modalities provide complementary information or that their joint use improves predictive performance. Therefore, the gap is interpreted alongside the complete-input Image + Text results and the individual unimodal results.

### 3.10. Asymmetric Loss and Uncertain-Label Masking

The 14-label classification problem is strongly imbalanced, with substantial variation in positive-label frequency and a large number of negative observations. ASL was therefore used in Experiments B–E as an established objective for long-tailed multi-label classification [26]. For binary target y and predicted probability p, Asymmetric Loss was defined in Equation (14).

(14) $$l_{\mathrm{ASL}}=-y(1-p{)}^{\gamma_{+}}\log(p)-(1-y)\left(p_{m}{)}^{\gamma_{-}}\log(1-p_{m}\right)$$

where γ+ and γ− are the focusing parameters for positive and negative samples, respectively, and pm denotes the margin-adjusted probability used for the negative component. The experiments used γ− = 4.0, γ+ = 1.0, and m = 0.05. Uncertain labels were excluded through a binary validity mask as shown in Equation (15).

(15) $$M_{\mathrm{ic}}=\left\{\begin{array}{c} 1,y_{\mathrm{ic}}\in \{0,1\},\\ 0,y_{\mathrm{ic}}=-1 \end{array}\right.$$

where M_ic_ is the validity indicator for sample ii and class c, y_ic_ is the corresponding target label, and uncertain labels encoded as -1 receive zero weight. The resulting masked optimization objective was as shown in Equation (16).

(16) $$L=\frac{\sum_{i=1}^{N}\sum_{c=1}^{C}M_{\mathrm{ic}}l\left(y_{\mathrm{ic}},p_{\mathrm{ic}}\right)}{\sum_{i=1}^{N}\sum_{c=1}^{C}M_{\mathrm{ic}}}$$

where N denotes the number of samples, C = 14 is the number of thoracic observations, p_ic_ is the predicted probability for class c of sample i, and M_ic_ ensures that uncertain labels do not contribute to optimization. Experiment A retained masked BCE to represent the original conventional baseline, whereas Experiments B–E used ASL.

### 3.11. Experimental Configuration

All controlled multimodal experiments followed the same underlying protocol wherever applicable. The optimizer was AdamW, with separate learning rates for pretrained encoders and newly initialized fusion/classification components. Model selection was based exclusively on validation Macro AUROC. The principal implementation, optimization, and training settings used across the controlled multimodal experiments are summarized in Table 4.

The maximum number of epochs acted as an upper training limit; the checkpoint obtaining the highest validation Macro AUROC was retained for subsequent evaluation. Threshold Selection and Evaluation under Modality Removal. Class-specific classification thresholds were selected using validation predictions obtained with complete Image + Text inputs and were retained during independent-test evaluation under Image + Text, Image-Only, and Text-Only conditions. This protocol maintains a consistent decision rule across the three inference conditions and prevents the independent test set from being used for threshold selection. However, removing one modality can shift the predicted-probability distributions, potentially making thresholds selected under complete-input conditions suboptimal for unimodal classification. Consequently, the resulting unimodal F1 scores reflect performance under the fixed complete-input thresholds rather than the best achievable F1 under modality-specific threshold optimization. A complementary analysis using thresholds selected independently for each unimodal condition from validation predictions can assess the sensitivity of F1 to this distribution shift, while preserving the independent test set for final evaluation.

### 3.12. Explainability Analysis

To complement the modality-ablation analysis, post hoc explainability was applied to the trained proposed framework using a representative Pleural Effusion-positive case from the independent test set. All analyses used the retained trained checkpoint without additional training or test-set threshold optimization. Grad-CAM was used to visualize class-specific spatial attribution under Image + Text and Image-Only inference. Because localization annotations were unavailable, the resulting maps were interpreted qualitatively rather than as quantitative disease-localization results. Integrated Gradients (IG) was applied to the PubMedBERT token-embedding layer under Text-Only inference to estimate token-level contributions to the Pleural Effusion prediction. IG was evaluated using 20, 50, and 100 integration steps, with convergence and attribution stability used to select the final configuration. The adaptive fusion gate was additionally examined to characterize latent modality weighting. Gate statistics were interpreted as representation-level weighting rather than direct percentages of visual or textual contribution.

## 4. Results

The standalone Swin-Tiny visual classifier was first evaluated to establish the discriminative capability available from chest radiographs independently of the textual modality. The model achieved a validation Macro AUROC of 0.7902 and an independent-test Macro AUROC of 0.7896, corresponding to a difference of only 0.0006 between the two held-out partitions. This stable performance confirms that the radiographic modality itself contains substantial predictive information for the 14 thoracic observations. Consequently, weak Image-Only performance following multimodal training cannot be attributed solely to insufficient information in the chest radiographs. The independent-test per-class performance of the standalone visual model is reported in Table 5.

At the class level, the strongest visual discrimination was observed for Pleural Effusion (AUROC = 0.8950), Support Devices (0.8748), Edema (0.8739), and No Finding (0.8609), whereas Fracture (0.5782) showed the lowest AUROC. PR-AUC varied more substantially across observations, ranging from 0.0375 for Fracture to 0.7582 for No Finding, reflecting the effect of class imbalance on precision–recall performance.

Five controlled multimodal configurations were subsequently evaluated using identical patient-wise partitions, Swin-Tiny and PubMedBERT encoders, a 512-dimensional projection space, and the same evaluation protocol. These experiments were designed to distinguish the effects of imbalance-aware optimization and fusion architecture from those of modality-balanced training. All configurations were evaluated under Image + Text, Image-Only, and Text-Only inference conditions. The resulting Macro AUROC, Macro PR-AUC, and Macro F1 scores are reported in Table 6.

**Figure 4:**
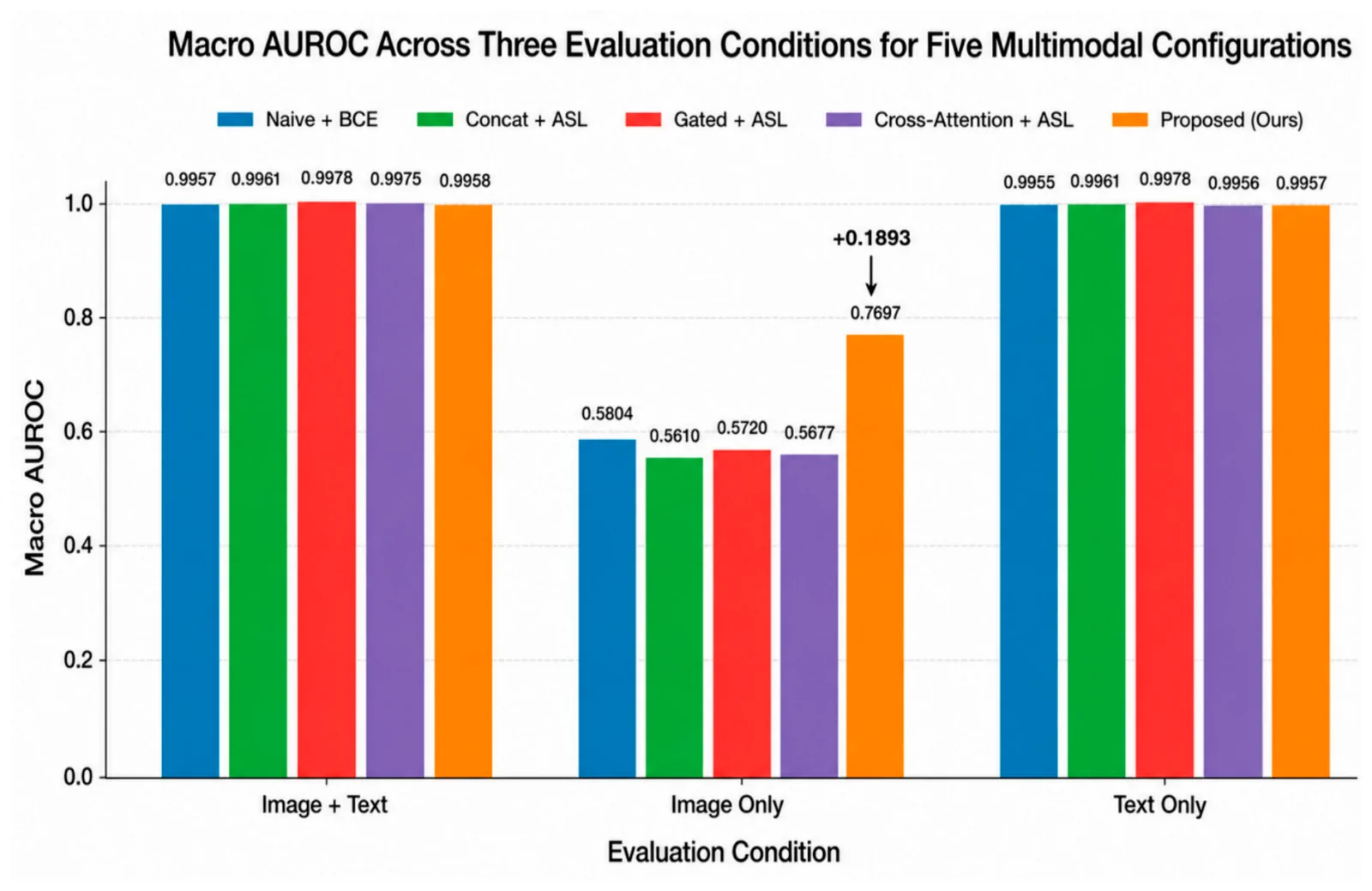
Macro AUROC for the five controlled multimodal configurations.

All five configurations achieved near-saturated performance when both modalities were available. The highest complete-input Macro AUROC was obtained by Gated + ASL (0.9978), followed by Cross-Attention + ASL (0.9975), while the proposed framework achieved 0.9958. A substantially different pattern emerged when the textual modality was removed. The Image-Only Macro AUROC decreased to 0.5804 for Naive + BCE, 0.5610 for Concat + ASL, 0.5720 for Gated + ASL, and 0.5677 for Cross-Attention + ASL. In contrast, the proposed framework retained an Image-Only Macro AUROC of 0.7697, an absolute improvement of 0.1893 over the naive baseline. The corresponding Macro PR-AUC increased from 0.1814 to 0.3428. Text-Only performance, however, remained almost identical to complete-input performance across the configurations. The comparative behavior of the five models under the three inference conditions is illustrated in Figure 4.

These findings demonstrate that neither replacing BCE with ASL nor introducing adaptive gating or cross-attention was sufficient by itself to preserve a strong visual pathway. Despite their high complete-input performance, the corresponding Image-Only AUROC values remained close to those of the naive baseline. The ablation results provide further insight into the contributions of the individual design choices. Comparing concatenation with BCE against concatenation with ASL examines the effect of the loss configuration, while comparing concatenation with ASL against adaptive gated fusion with ASL evaluates the effect of the fusion mechanism while retaining ASL for long-tailed multi-label learning. Furthermore, comparing gated fusion with ASL against the proposed model provides evidence regarding the contribution of modality dropout, provided that the remaining training conditions are comparable. These results suggest that the improvement in visual-pathway performance is associated with the complete proposed configuration rather than with the use of ASL or adaptive gating alone. However, the current comparisons do not fully isolate all component interactions, and the observed differences should therefore be interpreted in the context of the evaluated configurations. The principal improvement of the proposed framework therefore occurred not in complete-input discrimination, but in the amount of visual information retained after text removal. The same tendency was observed in the available micro-level evaluation: the proposed framework achieved an Image-Only Micro AUROC of 0.8788, Micro PR-AUC of 0.5880, and Micro F1 of 0.3289, compared with 0.6943, 0.2785, and 0.0033, respectively, for the naive baseline.

To quantify this asymmetric dependence more explicitly, text-removal degradation was calculated as the reduction in Macro AUROC from Image + Text to Image-Only inference, while image-removal degradation was calculated from Image + Text to Text-Only inference. The difference between these two degradations was defined as the modality-dominance gap. The resulting measurements are reported in Table 7.

The independently trained visual model provides an important reference for interpreting this reduction. Its test Macro AUROC of 0.7896 was only 0.0199 higher than the proposed model’s Image-Only AUROC of 0.7697. In comparison, the naive, Concat + ASL, Gated + ASL, and Cross-Attention + ASL configurations were lower than the standalone visual control by 0.2092, 0.2286, 0.2176, and 0.2219, respectively. Thus, modality-balanced training recovered most of the discriminative capability available to the independently optimized visual encoder. Importantly, Gated + ASL still achieved a higher complete-input Macro AUROC than the proposed framework (0.9978 vs. 0.9958) but retained substantially weaker Image-Only discrimination (0.5720 vs. 0.7697). This result demonstrates that maximizing complete-input multimodal performance does not necessarily imply balanced utilization of the constituent modalities.

To assess whether the modality-balancing behavior was specific to the dropout probabilities adopted in the primary proposed framework, a post hoc sensitivity analysis was conducted using alternative asymmetric modality-dropout configurations. The primary configuration used image- and text-modality dropout probabilities of 0.15 and 0.30, respectively. Additional configurations included a reversed asymmetric setting and a nearby alternative setting. These experiments were intended to examine the robustness of the observed modality-dependence pattern and were not used to retrospectively select the primary configuration. All configurations were evaluated using the same independent-test protocol under Image + Text, Image-Only, and Text-Only inference conditions shown in Table 8.

Table 8 compares the effects of different modality-dropout configurations on complete-input performance and visual discriminative capability. The primary proposed configuration, with image-dropout and text-dropout probabilities of 0.15 and 0.30, respectively, achieved an Image + Text Macro AUROC of 0.9958 and an Image-Only Macro AUROC of 0.7697, resulting in a modality-dominance gap of 0.2260. Reversing the dropout probabilities (0.30 for images and 0.15 for text) increased the Image-Only Macro AUROC to 0.7750 and reduced the dominance gap to approximately 0.2222. The alternative asymmetric configuration (0.20 image dropout and 0.30 text dropout) achieved the highest Image + Text Macro AUROC (0.9975) and Image-Only Macro AUROC (0.7859), together with the smallest dominance gap (approximately 0.2116) and the smallest performance drop when text was removed. In contrast, the performance drop when images were removed was close to zero across the evaluated configurations, indicating that complete-input AUROC remained largely unchanged when the visual modality was unavailable. Overall, these results suggest that the selected dropout probabilities influence visual performance retention and modality dependence, while the near-saturated complete-input AUROC alone does not demonstrate balanced use of both modalities. The sensitivity results should therefore be interpreted jointly using complete-input performance, the performance drop under text removal, and the modality-dominance gap.

**Figure 5:**
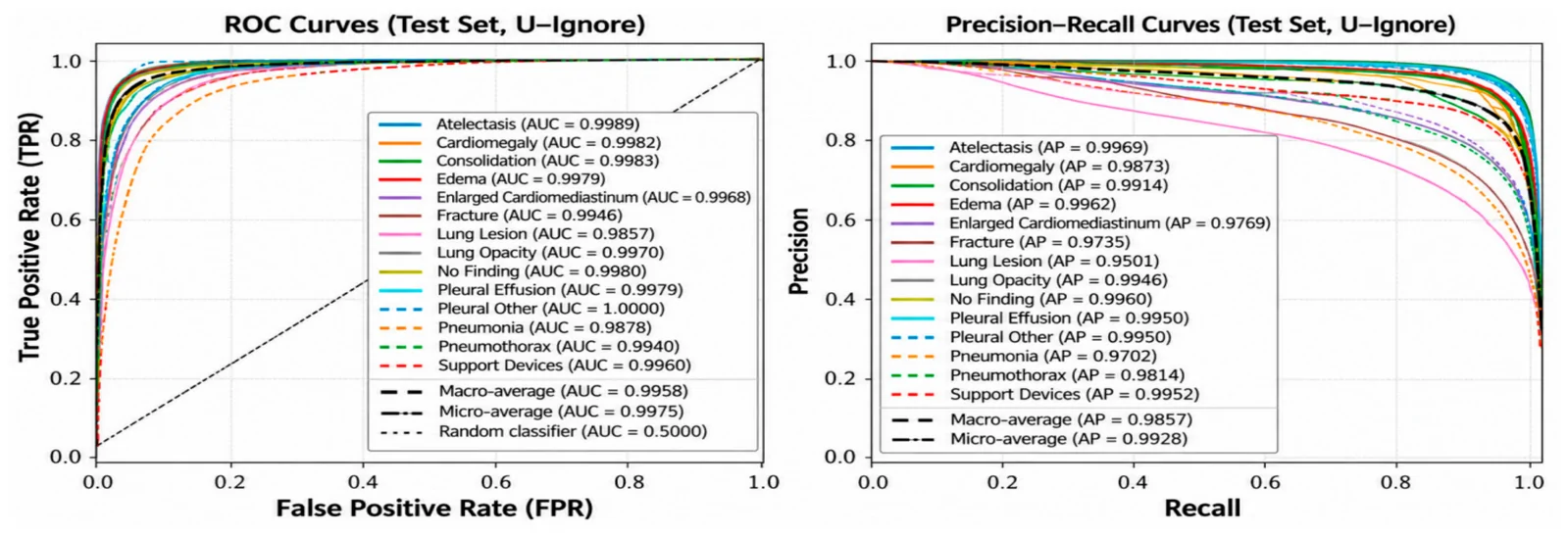
Per-class AUROC and PR-AUC of the proposed modality.

**Figure 6:**
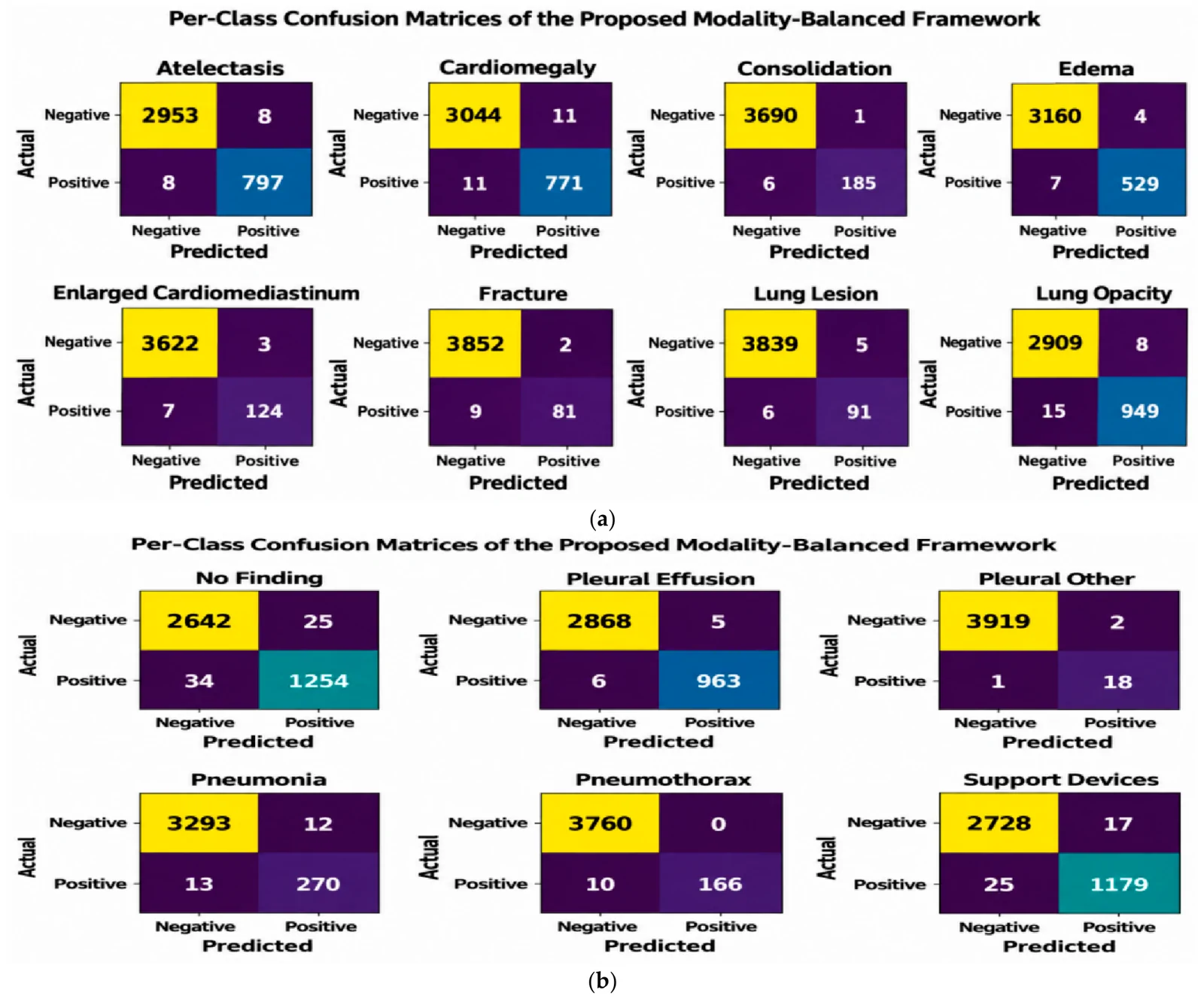
Per-class confusion matrices of the proposed modality-balanced framework on the independent test set: (**a**) Atelectasis, Cardiomegaly, Consolidation, Edema, Enlarged Cardiomediastinum, Fracture, Lung Lesion, and Lung Opacity; (**b**) No Finding, Pleural Effusion, Pleural Other, Pneumonia, Pneumothorax, and Support Devices.

Finally, the proposed framework was examined at the class level on the independent test set. Per-class AUROC and PR-AUC were used to characterize differences in discrimination across the 14 thoracic observations, with PR-AUC providing a complementary assessment under the long-tailed label distribution. The resulting class-specific performance is presented in Figure 5. The class-wise AUROC and PR-AUC results in Figure 5 provide a more detailed assessment of the proposed framework across the 14 thoracic observations than aggregate Macro metrics alone. PR-AUC is particularly relevant for the long-tailed label distribution because it provides complementary information about precision–recall performance, especially for low-prevalence findings. Particular attention should be given to Fracture, Lung Lesion, and Pleural Other, for which performance should be interpreted using their individual PR-AUC values rather than inferred from the overall Macro AUROC. In addition, precision, recall, and F1 under the fixed validation-derived thresholds provide complementary information about threshold-dependent classification performance. Therefore, the per-class results should be interpreted alongside the confusion matrices in Figure 6 to obtain a more comprehensive understanding of the proposed framework’s performance on rare findings.

The class-level analysis complements the aggregate Macro results by showing that overall performance is not distributed uniformly across thoracic findings. ROC and precision–recall curves were additionally examined to characterize class-specific ranking behavior, while per-class confusion matrices evaluated threshold-dependent prediction outcomes using the fixed validation-derived thresholds. These analyses are particularly relevant for low-prevalence observations, for which AUROC alone may provide an incomplete characterization of predictive performance. To further examine threshold-dependent classification performance, per-class confusion matrices were generated for the proposed framework on the independent test set using the fixed validation-derived thresholds. These matrices provide complementary information to the threshold-independent AUROC and PR-AUC measures by showing the distribution of true-positive, false-positive, true-negative, and false-negative predictions for each thoracic observation. The resulting confusion matrices are presented in Figure 6a,b.

To provide case-level interpretability for the quantitative modality-dependence findings, a representative Pleural Effusion-positive sample from the independent test set was examined using visual and textual attribution methods. The proposed model produced a Pleural Effusion probability of 0.9995 under complete Image + Text inference and 0.9994 under Text-Only inference, whereas the Image-Only probability decreased to 0.6558. With the frozen validation-derived threshold of 0.8034, the sample was classified as positive under Image + Text and Text-Only inference but negative under Image-Only inference. Thus, removal of the visual modality produced almost no change in prediction confidence for this case, whereas removal of the textual modality substantially reduced the prediction probability.

**Figure 7:**
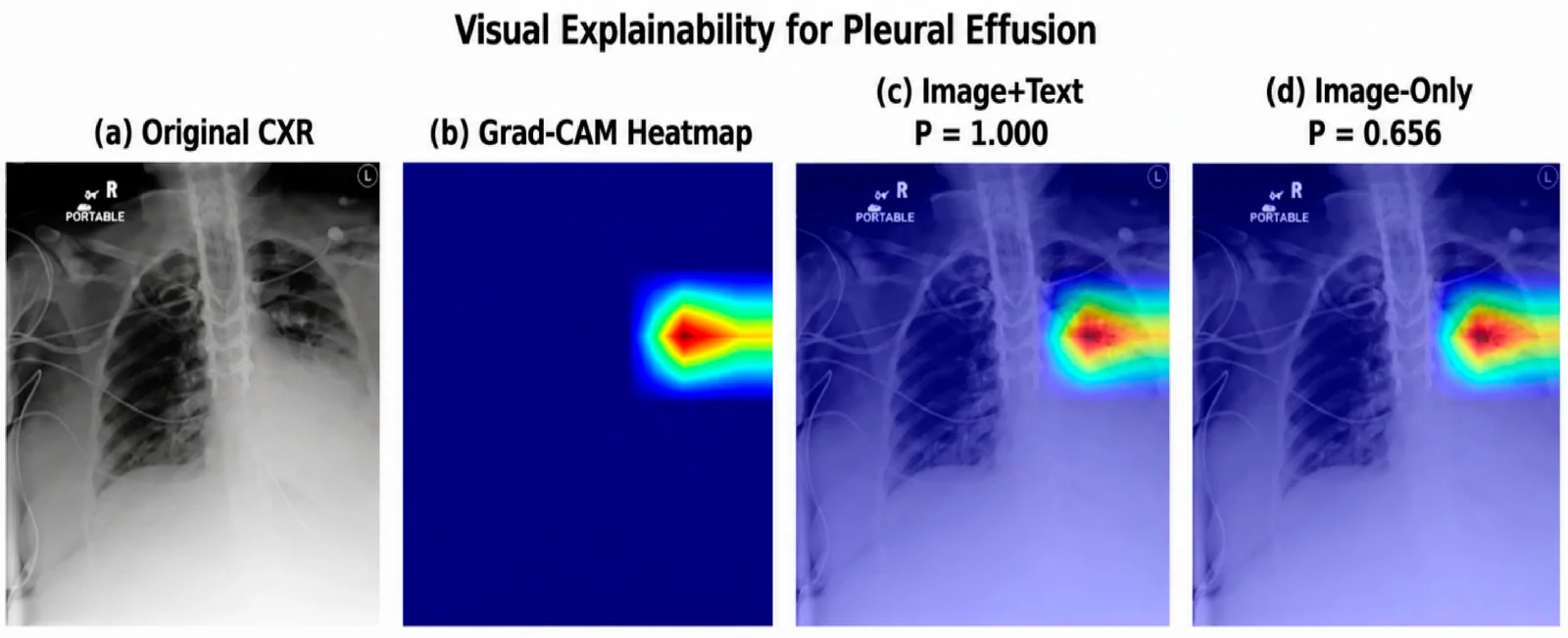
Visual explainability analysis for a representative Pleural Effusion-positive test sample.

As illustrated in Figure 7, Grad-CAM produced localized thoracic activation under both complete Image + Text and Image-Only inference. Although the Image-Only prediction confidence was substantially lower than that obtained with complete multimodal input, the presence of spatially localized activation indicates that the visual pathway retained structured radiographic information rather than becoming completely inactive. This observation is consistent with the aggregate Image-Only results, where modality-balanced training substantially improved visual-pathway discrimination compared with the other multimodal configurations. The numerical reliability and stability of the textual attribution were examined before interpreting the token-level explanation. Integrated Gradients was evaluated using 20, 50, and 100 integration steps. The corresponding convergence results are summarized in Table 9.

The 50-step configuration achieved the smallest absolute convergence delta (0.0176) and relative completeness error (0.00193) and was therefore selected for the final textual attribution visualization. The resulting explanations were also highly stable across integration settings. Token-level attribution correlations were 0.9837 between 20 and 50 steps, 0.9855 between 50 and 100 steps, and 0.9831 between 20 and 100 steps. Furthermore, 19 of the 20 highest-attribution tokens were shared in each pairwise comparison, corresponding to a 95% Top-20 overlap. These results indicate that the principal token-attribution pattern was stable across the evaluated integration settings.

**Figure 8:**
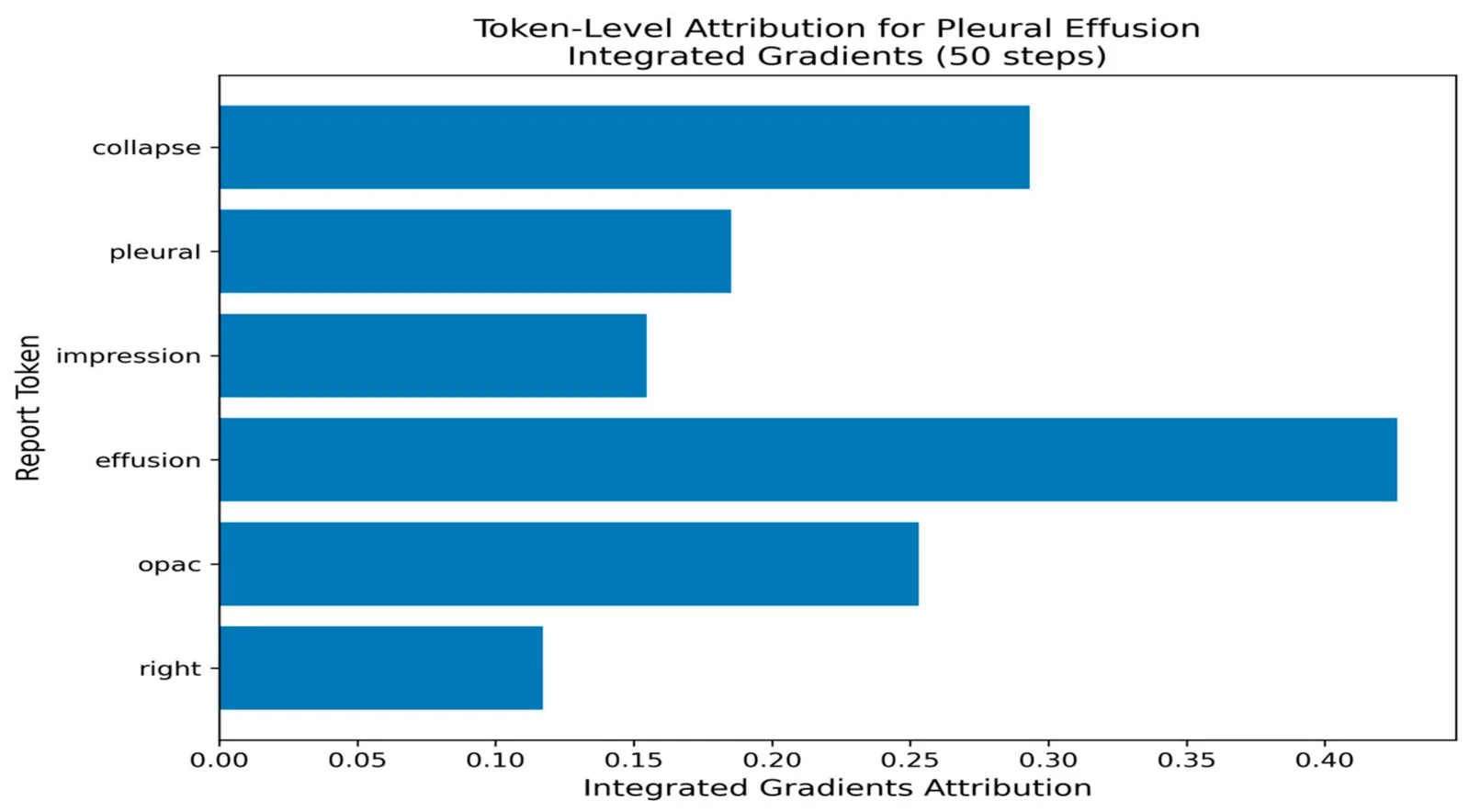
Integrated Gradients-based textual attribution for Pleural Effusion in a representative test report.

As shown in Figure 8, the textual pathway assigned strong positive attribution to clinically relevant terms associated with the target finding, including “effusion,” “pleural,” “opacity,” and “collapse.” Analysis of repeated token occurrences further showed that “effusion” had the largest aggregated positive attribution (1.1047), followed by “pleural” (0.6527), “opacity” (0.5384) and “collapse” (0.4609). In contrast, “pneumothorax” exhibited a negative aggregated attribution of −0.2279, consistent with the explicit negation of Pneumothorax in the corresponding report. The attribution pattern therefore demonstrates that the textual pathway strongly exploited explicit disease-related terminology associated with the target observation. The adaptive fusion gate further supported the observed modality asymmetry. Under Image + Text inference, the mean and median gate values were 0.1027 and 0.0044, respectively. According to Equation (12), lower gate values correspond to greater weighting of textual features, indicating strong textual weighting for this case. Because gating operates element-wise across the 512-dimensional representation, these values should be interpreted as latent feature weights rather than direct percentages of modality contribution. Overall, the experiments reveal a clear distinction between complete-input predictive performance and modality-specific robustness. Although all controlled multimodal configurations achieved near-saturated Image + Text performance, conventional concatenation, ASL, adaptive gating, and cross-attention retained weak Image-Only discrimination. The proposed modality-balanced framework was evaluated for performance improvement over the naive baseline by increasing Image-Only Macro AUROC to 0.7697 and by narrowing the gap by around 45.6% compared to the naive baseline, while keeping Image + Text Macro AUROC high at 0.9958. These quantitative results were further supported by a case-level explainability analysis: Grad-CAM indicated that the proposed framework could capture the spatially structured visual information, while Integrated Gradients and gate analysis indicated that they could capture explicit textual information well. However, Text-Only performance was nearly as similar to complete-input performance. All of these results suggest that the proposed framework could significantly enhance visual-pathway preservation and reduce, but not eliminate, text dominance.

## 5. Discussion

The results show that high MODAL CLASSIFICATION performance does not necessarily reflect the balanced use of images and text. All tested configurations performed well on the Image + Text Macro AUROC (>0.995), but a significant performance drop was observed when the text modality was omitted. The Text-Only Macro AUROC of the naive model was 0.9955, while the Image-Only inference result was 0.5804, and the results of the other controls were similarly low. The results are in line with the results of the previous work that indicates that multimodal models can focus on a more powerful or more accessible predictive modality [11,12,13,14,15,16,17]. In the present CXR–report setting, radiology reports provide highly direct disease-related information, making the textual pathway substantially easier to exploit than learning the same abnormalities from image pixels. The controlled experiments further indicate that neither imbalance-aware loss nor more expressive fusion was sufficient to resolve this dependence. Replacing masked BCE with ASL slightly improved complete-input performance but did not improve Image-Only discrimination, while adaptive gated and cross-attention fusion also retained substantial text dependence despite achieving very high complete-input AUROC. ASL remains valuable for addressing positive–negative and inter-class imbalance in multi-label classification [26,27,28,29]; however, the present findings suggest that label imbalance and modality imbalance are distinct problems. The former can be addressed through loss design, whereas the latter requires explicit mechanisms that encourage utilization of individual modalities.

The principal improvement emerged after introducing training-time modality dropout. The proposed framework achieved an Image-Only Macro AUROC of 0.7697, compared with 0.5720 for Gated + ASL without modality dropout, while maintaining a complete-input Macro AUROC of 0.9958. Its modality-dominance gap decreased to 0.2260, representing an approximately 45.6% reduction relative to the naive baseline. This behavior is consistent with previous work showing that modality dropout and missing-modality training can improve robustness by preventing persistent reliance on a single modality [19,20,21,22,23,24,25]. Importantly, the independently trained Swin-Tiny achieved a test Macro AUROC of 0.7896, only 0.0199 above the proposed model’s Image-Only performance. This suggests that modality-balanced training recovered most of the visual discriminative capability that conventional multimodal optimization failed to preserve. Nevertheless, the proposed framework mitigated rather than eliminated text dominance. Text-Only Macro AUROC remained 0.9957, almost identical to the complete-input value of 0.9958, while Image-Only performance remained lower at 0.7697. Equal contribution from both modalities should not necessarily be expected because radiographs and their associated reports contain different levels and forms of diagnostic information. The comparatively low Image-Only Macro F1 of 0.1367 should also be interpreted in the context of the frozen-threshold protocol; thresholds were selected from complete Image + Text validation predictions and were not recalibrated following modality removal. Because ablation can alter prediction-score distributions, threshold-dependent F1 may decrease substantially even when useful ranking information is retained. Macro AUROC therefore provides the primary measure of visual-pathway retention in this analysis.

The explainability analysis provides qualitative, case-level illustrations of mechanisms that may contribute to the observed modality dependence. In the representative case examined, Grad-CAM highlighted spatially localized regions in the chest radiograph, while Integrated Gradients assigned strong attribution to report terms such as “effusion” and “pleural.” The observations here reflect the response of the visual and textual pathways under the example selected, but do not provide evidence for whether this is the case for the entire dataset or whether the highlighted regions represent clinically validated disease sites. Thus, these visualizations are viewed as complementary evidence to the quantitative modality-ablation results and are not used to confirm results of the quantitative modality-ablation on their own. Further patient and thoracic analyses and systematic stability evaluations of the attribution should be conducted to confirm these patterns. However, a Text-Only Macro AUROC score of 0.9955 should be interpreted with caution as well. The observed performance with only the text input may partly be due to the overlap between the text input and the structured target labels, as the input may include diagnostic information. In the present study, however, the extent of this contribution is not quantified, and the high AUROC by itself does not prove that this leakage of the target has taken place. To test this more directly, further investigation into report context controls and leakage-sensitive evaluation procedures is needed. This concern is especially important since the thoracic observation labels used in this study come from radiology reports, which results in a close relationship between the text input and the supervision source. Thus, the near-saturated Text-Only performance should not be seen as proof that text diagnostics are intrinsically better than those from radiography. Rather, it emphasizes a context where the term of the report can be a very direct predictive signal that can lead the model to excessively weight text information for multimodal optimization. Additional qualitative evidence of feature-level weighting towards text-based representations was obtained by analyzing the adaptive fusion gate, where both modalities were available. Gate values should not be interpreted as percentages of modality contribution, or causal measures of modality importance because the gate is element-wise applied to the latent representation. Moreover, observations from the representative explainability case should not be extended to the entire dataset without systematic multi-case validation. The quantitative ablation findings, combined with the qualitative explainability studies, call for considering the performance of medical VLM rather than exhaustive input performance. Unimodal controls, modality-dependence measures, targeted modality ablation, and complementary explainability analyses may be useful to uncover whether good overall performance has been at the expense of underutilization of the individual modality. The present results suggest that modality-balanced training is effective at enhancing the discrimination of visual-pathway stimuli while maintaining high complete-input performance under the conditions tested. However, additional research is warranted to validate these enhancements, to ensure the stability of the attribution patterns and their clinical relevance, and the degree of meaningful cross-modal complementarity the framework provides. While there is a significant improvement in preserving visual pathways, there are a few things that need to be taken into account. First, all experiments were performed on MIMIC-CXR/MIMIC-CXR-JPG [1,30]. Patient-level splitting ensured that there was no overlap between training, validation, and independent test sets, but external validation is needed to see if the modality dependence observed and the effectiveness of the proposed balancing strategy extend beyond the institution, imaging system, patient population, and reporting style. Second, the report from the radiology department always contains descriptions that are highly informative about the CXR and can be used as labels. This accounts for some of the extraordinarily powerful textural pathway. Text-Only Macro AUROC was almost as high as the complete-input value (0.9957), despite modality-balanced training. Therefore, the proposed framework mitigates rather than eliminates text dominance. Future studies could investigate settings in which textual inputs are less directly aligned with the prediction targets, thereby providing a more stringent evaluation of cross-modal complementarity. Third, although several controlled configurations were evaluated, including Concat + BCE, Concat + ASL, Gated + ASL, Cross-Attention + ASL, and the proposed modality-balanced framework, the individual and interaction effects of adaptive gated fusion, modality dropout, and Asymmetric Loss (ASL) were not exhaustively isolated through a complete set of controlled experiments. The existing comparisons provide useful evidence regarding alternative loss and fusion configurations, as well as the potential contribution of modality dropout when the remaining training conditions are comparable. However, additional controlled ablations are needed to quantify the independent and interactive contributions of these components while keeping other training conditions fixed. Fourth, statistical uncertainty and reproducibility remain limitations of the current evaluation. The reported performance values are point estimates obtained under the evaluated experimental configurations. Repeated training runs with different random seeds were not conducted; therefore, variability across independent training runs and the statistical significance of the observed performance differences have not been established. Although the proposed framework achieved a higher Image-Only Macro AUROC than the conventional baseline, this improvement should be interpreted as an empirical result under the current experimental configuration rather than a statistically confirmed effect. Future studies should involve several independent training sessions and report the mean performance, standard deviations, and a suitable confidence interval or paired statistical tests to evaluate consistency and statistical significance of observed gains. Fifth, class-specific thresholds were optimized with full Image + Text validation predictions, and then were fixed for modality-removal evaluation. This offers a uniform robustness test, but removing a modality alters the distribution of prediction scores, which may explain the difference between ablated AUROC and F1. Future studies need to explore the calibration and threshold selection of each modality based on the validation data, and the independent test set should not be modified in the model selection process. Sixth, explainability analysis was only conducted on an ad hoc basis on individual cases. These findings should therefore be considered as additional evidence of disease localization and/or of the contribution of disease modality rather than as quantitative evidence of disease localization or of the contribution of disease modality. Grad-CAM gives qualitative spatial attention information, but it is difficult to determine if the regions of interest that are highlighted correspond to clinically annotated lesions. Likewise, Integrated Gradients and adaptive-gate values refer to input attribution and learned feature weighting, respectively, but do not directly measure modality importance. These analyses should be expanded to a larger patient population and to a wider spectrum of thoracic abnormalities and to a larger number of datasets with lesion locations annotated by experts. The quantitative assessment of stability of attribution, faithfulness of the representation of the information, and interactions between modalities would give a better understanding of the use of the visual information and the text. Finally, the evaluation was restricted to paired chest radiographs and radiology reports from MIMIC-CXR/MIMIC-CXR-JPG, limiting the conclusions that can be drawn about clinical applicability and generalization across institutions. In clinical practice, radiology reports may be unavailable during image interpretation, incomplete, noisy, or written according to reporting conventions that vary between institutions. When reports are unavailable, predictions must rely on visual information alone. The observed Image-Only performance therefore provides relevant evidence of the model’s ability to retain discriminative information without textual input. However, the gap in performance between Text-Only and Image-Only conditions suggests the framework still has quite a bit of dependence on textual information. The current study did not specifically address the impact of incomplete or incomplete (but not noisy) reports, or the differences in reporting, on predictions. Patients may also differ in terms of their disease prevalence, imaging equipment, and clinical workflows from one institution to another, which can also influence performance. This framework could be tested with reports that are missing or intentionally altered and tested with other clinical institutions to validate its performance in the future. These assessments should be performed on class-wise discrimination, threshold-dependent measures, and robustness to distribution shifts, comparing the performance of Image + Text with that of Image-Only. The framework could also be explored through further research examining other clinical modalities, other forms of balancing, disease-specific balancing strategies, uncertainty-aware modality weighting, and cross-modal complementarity, to better understand the generalizability and potential clinical utility of the framework.

## 6. Conclusions

This study explored text dominance in the multi-label chest X-ray vision–language classification task, which is an inherently multi-label classification problem. The results showed that the performance in multimodal tasks was not necessarily matched by successful use of visual information. The naive fusion baseline achieved a Macro AUROC of 0.9957 with Image + Text input, but its Macro AUROC decreased to 0.5804 when evaluated using images alone. Text-Only performance remained high at 0.9955. Changes to the loss function and the use of more complex fusion methods were insufficient to resolve this imbalance. Concat + ASL, Gated + ASL, and Cross-Attention + ASL also showed low Image-Only performance despite strong results with both modalities. These findings highlight the difference between achieving high multimodal accuracy and making effective use of visual information. To address this issue, the proposed framework combined adaptive gated fusion, training-time modality dropout, and ASL for long-tailed classification. It achieved an Image + Text Macro AUROC of 0.9958 and improved Image-Only Macro AUROC to 0.7697. Compared with naive fusion, this represents an absolute improvement of 0.1893, while the modality-dominance gap decreased from 0.4151 to 0.2260, corresponding to an approximately 45.6% reduction in asymmetric modality dependence. The independently trained visual model achieved a Macro AUROC of 0.7896, further demonstrating that substantial predictive information existed in the CXR modality but was underutilized during conventional multimodal optimization. The proposed model recovered most of this visual capability, although text remained the dominant modality. Complementary explainability analysis using Grad-CAM, Integrated Gradients, and adaptive-gate inspection further supported these findings by showing retained spatially structured visual evidence together with strong reliance on explicit disease-related textual information. Overall, the findings demonstrate that multimodal performance and multimodal utilization should be evaluated separately. High Image + Text performance alone cannot establish whether a model meaningfully utilizes all available modalities. Combining standalone unimodal controls, targeted modality ablation, modality-dependence measures, and complementary explainability analysis therefore provides a more informative evaluation of medical vision–language systems and can guide the development of models that are not only highly predictive but also more robust to asymmetric modality dependence.

## Figures and Tables

**Table 1:** Comparison of existing studies with the proposed framework.

Study	Main Approach	Main Contribution	Difference from the Proposed Study
Zhang et al. (2022)—ConVIRT [5]	Bidirectional contrastive learning between medical images and paired text	Learns medical visual representations from image–text pairs and improves data efficiency in medical image recognition.	Focuses on contrastive representation learning rather than explicitly reducing text dominance in long-tailed multi-label CXR classification.
Huang et al. (2021)—GLoRIA [6]	Global–local image–text contrastive learning	Aligns image subregions with words in radiology reports to learn label-efficient medical representations.	Emphasizes global–local representation learning, whereas the proposed study targets asymmetric modality dependence and visual-pathway retention.
Ridnik et al. (2021)—Asymmetric Loss (ASL) [4]	Asymmetric Loss for multi-label classification	Down-weights easy negative examples and addresses positive–negative label imbalance.	Addresses label imbalance but does not, by itself, directly address the imbalance between textual and visual modalities.
Gu et al. (2025) [9]	Contrastive multimodal fusion with improved modality dropout	Improves robustness to missing modalities using learnable modality tokens, modality dropout, and multimodal contrastive learning.	Focuses on modality missingness and multimodal robustness; the proposed study specifically investigates text dominance in paired CXR images and reports.
Wu et al. (2025) [14]	Analysis of text dominance in multimodal large language models	Investigates modality dominance across multiple data modalities and proposes metrics and token compression to rebalance attention.	Studies general multimodal large language models rather than the specific problem of long-tailed multi-label CXR classification using dedicated visual and textual encoders.
Zeng et al. (2026) [13]	Prior-aware multi-label CXR classification with prototype-based contrastive learning	Uses pathology relationships, label-aware feature decoupling, and prototype contrastive learning to improve discrimination among pathologies.	Focuses on pathology representation and classification performance rather than directly measuring and mitigating text dominance.
Proposed study	Adaptive gated fusion, ASL, and training-time modality dropout	Evaluates Image-Only, Text-Only, and Image + Text performance to investigate asymmetric modality dependence while strengthening visual-pathway retention.	Targets text dominance in long-tailed multi-label CXR classification and evaluates both modality-specific performance and multimodal discrimination.

**Table 2:** Dataset characteristics and experimental partitions.

Partition	Samples	Patients	Purpose
Training	32,942	12,503	Parameter optimization
Validation	4024	1563	Model and threshold selection
Independent test	3955	1563	Final evaluation
Total	40,921		

**Table 3:** Multimodal experimental configurations.

ID	Model	Fusion Strategy	Loss	Training-Time Modality Dropout	Experimental Purpose
A	Conventional baseline	Concatenation	Masked BCE	No	Original multimodal baseline
B	Concat + ASL	Concatenation	ASL	No	Effect of imbalance-aware loss
C	Cross-Attention + ASL	Cross-attention	ASL	No	Strong interaction-based fusion
D	Gated + ASL	Adaptive gated fusion	ASL	No	Effect of learnable gating
E	Proposed modality-balanced model	Adaptive gated fusion	ASL	Yes	Modality-balanced training

**Table 4:** Experimental configuration used for controlled multimodal comparison.

Parameter	Setting
GPU hardware	NVIDIA GeForce RTX 3060
Visual encoder	Swin-Tiny
Text encoder	PubMedBERT
Software	Python 3.9.1, PyTorch 2.0.1, Torchvision 0.15.2,CUDA runtime 11.8 and Transformers 4.30.2
Random seed	42
Training duration(Purposed Model)	4 h 30 min
Proposed fusion strategy	Adaptive gated fusion
Classification task	Multi-label classification
Number of output labels	14 thoracic observations
Imbalance-aware loss	Asymmetric Loss (ASL)
ASL negative focusing parameter (γ−)	4.0
ASL positive focusing parameter (γ+)	1.0
ASL probability margin (m)	0.05
Proposed training strategy	Training-time modality dropout
Simultaneous removal of both modalities	Not permitted
Optimizer	AdamW
Encoder learning rate	1 × 10^−5^
Fusion/head learning rate	1 × 10^−4^
Weight decay	1 × 10^−4^
Batch size	4
Maximum training epochs	8
Early-stopping criterion	Validation Macro AUROC
Early-stopping patience	3 epochs

**Table 5:** Independent-test per-class performance of the standalone visual model.

Thoracic Finding	AUROC	PR-AUC	F1	Precision	Recall	Specificity
Atelectasis	0.7944	0.4739	0.5191	0.3993	0.7416	0.6967
Cardiomegaly	0.7794	0.4331	0.4963	0.3665	0.7685	0.6599
Consolidation	0.7687	0.1174	0.1856	0.1073	0.6859	0.7047
Edema	0.8739	0.5005	0.5440	0.4413	0.7090	0.8480
Enlarged Cardiomediastinum	0.6632	0.0637	0.0971	0.0583	0.2901	0.8306
Fracture	0.5782	0.0375	0.0772	0.0543	0.1333	0.9458
Lung Lesion	0.7027	0.0691	0.1037	0.0682	0.2165	0.9253
Lung Opacity	0.7447	0.4679	0.5153	0.3873	0.7697	0.5975
No Finding	0.8609	0.7582	0.7159	0.6549	0.7896	0.7990
Pleural Effusion	0.8950	0.7259	0.6847	0.5941	0.8081	0.8138
Pleural Other	0.7599	0.0408	0.0479	0.0254	0.4211	0.9217
Pneumonia	0.7103	0.1880	0.2265	0.1527	0.4382	0.7918
Pneumothorax	0.7701	0.1790	0.2137	0.1609	0.3182	0.9223
Support Devices	0.8748	0.7443	0.7123	0.6736	0.7558	0.8393

**Table 6:** Controlled comparison of multimodal configurations.

Model	Fusion/Training	Condition	Macro AUROC	Macro PR-AUC	Macro F1
Naive + BCE	Concatenation	Image + Text	0.9957	0.9774	0.9762
		Image-Only	0.5804	0.1814	0.0007
		Text-Only	0.9955	0.9775	0.9724
Concat + ASL	Concatenation	Image + Text	0.9961	0.9841	0.9676
		Image-Only	0.5610	0.1929	0.0477
		Text-Only	0.9961	0.9840	0.9660
Gated + ASL	Adaptive Gate	Image + Text	0.9978	0.9905	0.9750
		Image-Only	0.5720	0.2078	0.0587
		Text-Only	0.9978	0.9904	0.9750
Cross-Attention + ASL	Cross-Attention	Image + Text	0.9975	0.9902	0.9786
		Image-Only	0.5677	0.1731	0.0894
		Text-Only	0.9956	0.9842	0.9414
Proposed	Adaptive Gate + Modality Dropout + ASL	Image + Text	0.9958	0.9857	0.9699
		Image-Only	0.7697	0.3428	0.1367
		Text-Only	0.9957	0.9849	0.9695

**Table 7:** Macro AUROC modality-dependence analysis across the five multimodal configurations.

Model	Image + Text	Image-Only	Text-Only	Drop When Text Removed	Drop When Image Removed	Dominance Gap
Naive + BCE	0.9957	0.5804	0.9955	0.4153	0.0002	0.4151
Concat + ASL	0.9961	0.5610	0.9961	0.4351	≈0.0000	0.4351
Gated + ASL	0.9978	0.5720	0.9978	0.4258	≈0.0000	0.4258
Cross-Attention + ASL	0.9975	0.5677	0.9956	0.4298	0.0019	0.4279
Proposed	0.9958	0.7697	0.9957	0.2261	0.0001	0.2260

**Table 8:** Validation-based sensitivity analysis of modality-dropout configurations.

Configuration	Image Dropout	Text Dropout	Image + Text Macro AUROC	Image-Only Macro AUROC	Text-Only Macro AUROC	Drop When Text Removed	Drop When Image Removed	Dominance Gap
Naive baseline	0.00	0.00	0.9957	0.5804	0.9955	0.4153	0.0002	0.4151
Primary proposed	0.15	0.30	0.9958	0.7697	0.9957	0.2261	0.0001	0.2260
Reversed asymmetric	0.30	0.15	0.9973	0.7750	0.9972	0.2222	0.000015	0.222259
Alternative asymmetric	0.20	0.30	0.9975	0.7859	0.9975	0.2115	−0.000021	0.211613

**Table 9:** Integrated Gradients convergence analysis for the representative Pleural Effusion case.

IG Steps	Convergence Delta	Absolute Delta	Relative Completeness Error
20	1.0180	1.0180	0.1117
50	−0.0176	0.0176	0.0019
100	−0.3628	0.3628	0.0398

## Data Availability

Data available on request from the corresponding author.

## List of Abbreviations

Abréviation	Full form
AdamW	Adaptive Moment Estimation with Decoupled Weight Decay
ASL	Asymmetric Loss
AUROC	Area Under the Receiver Operating Characteristic Curve
BCE	Binary Cross-Entropy
BioViL	Biomedical Vision–Language Representation Learning
BioClinicalBERT	BERT adapted to clinical text
CXR	Chest X-ray
CNN	Convolutional Neural Network
Grad-CAM	Gradient-weighted Class Activation Mapping
IG	Integrated Gradients
LLM	Large Language Model
MHA	Multi-Head Attention
MIMIC-CXR	Medical Information Mart for Intensive Care—Chest X-ray
MIMIC-CXR-JPG	JPEG-based version of the MIMIC-CXR dataset
PR-AUC	Area Under the Precision–Recall Curve
ROC	Receiver Operating Characteristic
ASL γ+	Positive focusing parameter in Asymmetric Loss
ASL γ−	Negative focusing parameter in Asymmetric Loss
Swin-Tiny	Tiny variant of the Shifted-Window Transformer
U-Ignore	Uncertain-label ignoring strategy
VLM	Vision–Language Model
